# Psychosocial Determinants of Marital Satisfaction Among Gynecologic Cancer Survivors in Malaysia

**DOI:** 10.3389/fpsyt.2021.744922

**Published:** 2021-09-28

**Authors:** Sidi Hatta, Luke Sy-Cherng Woon, Nik Mohd Nor Nik Sumayyah, Shafiee Mohamad Nasir

**Affiliations:** ^1^Department of Psychiatry, Faculty of Medicine, National University of Malaysia, Kuala Lumpur, Malaysia; ^2^Department of Obstetrics and Gynecology, Faculty of Medicine, National University of Malaysia, Kuala Lumpur, Malaysia

**Keywords:** cancer survivors, educational achievement, gynecologic neoplasms, marital relationship, sexual dysfunctions

## Abstract

**Introduction:** Marital issues among gynecologic cancer survivors are common but complex. This study aimed to investigate the relationship between sociodemographic and clinical factors, including sexual dysfunction and marital satisfaction among Malaysian gynecologic cancer survivors.

**Methods:** A cross-sectional survey of married women with gynecologic cancers was conducted at a Malaysian university hospital. Sociodemographic and clinical data were gathered. Sexual dysfunction was measured using the Malay Version Female Sexual Function Index (MVFSFI), while marital satisfaction was evaluated with the Malay Version Golombok Rust Inventory for Marital Satisfaction (MVGRIMS).

**Results:** A total of 116 patients participated in this study. The median age was 59.0 years (Interquartile range, IQR: 49.0–67.0 years); the median duration of marriage was 32 years (IQR: 20.0–40.8 years). 80.2% had a secondary and lower level of education. 37.9% of study subjects (*n* = 44) reported poor-and below-levels of marital satisfaction, which was equivalent to MVGRIMS transformed scores of >5. The median FSFI total score was 49.9 (IQR: 2.0–63.0). MVGRIMS transformed score correlated significantly with all MVFSFI sub-scores. In logistic regression, lower educational levels were associated with poor marital satisfaction [primary, (adjusted Odds Ratio) aOR = 12.67, 95% CI: 1.40–114.87; secondary: aOR = 11.52, 95% CI: 1.39–95.72], while higher MVFSFI total score reduced the likelihood of poor marital satisfaction (aOR = 0.979, 95% CI: 0.964–0.994).

**Conclusion:** Both sexual dysfunction and low education level may affect marital satisfaction among gynecologic cancer survivors. Targeted efforts focusing on sex education for patients may help to improve marital satisfaction.

## Introduction

The diagnosis of gynecologic cancer and implications from the ensuing therapy profoundly alter the lives of women with cancers. Survivors of gynecologic cancers often encounter psychosocial issues, as manifested in the areas of group functioning, and role-taking, relationship with family, and marital relationship (1).

Marital issues among gynecologic cancer survivors are common. In a study, among women with gynecologic cancer who were in relationships, 27% encountered marital dysfunction (2). Such marital problems are often complex, involving various factors. For many survivors, sexual dysfunction issues seem to be the most distressing, as impaired sexual performance is perceived as jeopardizing their relationship with their partners (3). Many gynecologic cancer patients consciously keep their physical and emotional distance from their spouses to pre-empt possible rejection (4).

A recent study focusing on the relationship between sexual dysfunction and the quality of marital relationships in breast and genital cancer women found that recent sexual dysfunction had significantly poorer relationships with their spouses (5). Conversely, women who had a more intimate relationship with their partners experienced better sex life (6).

Sociodemographic factors are important contributing factors to the occurrence of gynecologic cancers. For instance, low education level, low-income level, and low occupation level (i.e., manual/unskilled labor) are risk factors of cervical cancer (7, 8). Moreover, sociodemographic factors also contribute to poor quality of life and psychosocial adjustment in gynecologic cancer survivors. Such factors include lower education, poor social support, and lower levels of religious belief (9). There is also evidence to suggest clinical factors such as treatment modalities may affect psychosocial adjustment in patients (10, 11).

Not many studies have been conducted in the Malaysian context to address issues related to marital satisfaction in a conservative Malaysian community (12). Indeed, to the best of our knowledge, no study was conducted on marital satisfaction among gynecological cancer survivors. Mustafa et al. outlined issues on the conventional values about relationships, emphasizing the stability of the relationship and belief in traditional gender role expectations in Malaysian middle-aged women. In their study, couples tend to spend their time together and avoid arguments and conflicts in their daily life (12). Sidi et al. found that discussing sex openly may be taboo but emphasized the importance of researching in this field of specialty to improve the betterment and quality of life among Malaysian women (13, 14). Sidi et al. reported that sexual topic is well-received in the suburban Malaysian society during their study on the validation of the Female Sexual Functioning Index (FSFI), especially at the face and content validity of the questionnaire (15).

The main objective of this study was to investigate the level of marital satisfaction among a group of gynecologic cancer survivors in Malaysia. The secondary objective was to explore the relationship between psychosocial and clinical factors, including sexual dysfunction, with marital satisfaction among gynecologic cancer survivors. We hypothesized that marital dissatisfaction was prevalent among gynecologic cancer survivors, and that it was associated with sexual dysfunction and other psychosocial determinants.

## Materials and Methods

This was a cross-sectional study. The study population was patients with gynecologic cancer who received treatment at the National University of Malaysia Medical Centre located at Bandar Tun Razak, Cheras in metropolitan Kuala Lumpur, Malaysia. The study subjects were recruited via convenience sampling from June 2017 to March 2020. The inclusion criteria were: (1) Malaysian citizens; (2) age of at least 18 years old; (3) married; (4) diagnosed of gynecologic cancer; and (5) had completed treatment for a minimum of 3 months. Disease progression on treatment and significant amnesia were the exclusion criteria. Sample size calculation was based on the estimated prevalence of marital dissatisfaction of 40% (16) and the study population of 160 (based on clinic records). Using the formula for sample size calculation for prevalence studies with finite population correction (precision = 0.05), the sample size required was 112.

Informed consent was obtained from all eligible participants before their participation. They were asked to fill up a self-administered questionnaire on sociodemographic data, including age, ethnicity, marital status, number of children, level of education, and employment status. Existing medical records were accessed to trace clinical data on the type and stage of cancer, duration of disease, type of treatment received, and duration of treatment completion. In addition, two validated instruments were employed to evaluate sexual dysfunction and marital satisfaction, respectively. Sexual dysfunction was measured using the Malay Version Female Sexual Function Index (MVFSFI), while marital satisfaction was evaluated with the Malay Version Golombok Rust Inventory for Marital Satisfaction (MVGRIMS).

The FSFI is a brief and reliable self-report measure of female sexual function developed by Rosen et al. (17). This questionnaire contains 19 items covering six domains of female sexual dysfunction, i.e., desire, subjective arousal, lubrication, orgasm, satisfaction, and pain. The lower the scores, the higher likelihood the women would suffer from sexual dysfunction. The Malay version of FSFI (MVFSFI) used in this study has been validated in the Malaysian population (15). The instrument demonstrated good face, content, and criterion validity. It also showed a high level of test-retest reliability. Regarding the reliability, the strongest correlation (0.973) was demonstrated for the domain of satisfaction in MVFSI and the weakest correlation for the domain of arousal (0.767). Total score ≤ 55 and above was found to be the suitable cut-off point to distinguish between women with sexual dysfunction and those without, with a sensitivity of 99% and a specificity of 97%.

The Golombok-Rust Inventory of Marital State (GRIMS) by Rust et al. is among the most used instruments in research to assess marital satisfaction (18). It is a 28-item structured questionnaire covering the degree of dependence and independence, warmth, love and hostility, trust and respect, coping with problems, and crisis. Each item has a four-point Likert scale, from score 0 for 'Strongly disagree' to score 3 for 'Strongly agree'. A total score is then computed (range: 0–84), with a high score indicating a likelihood of marital dissatisfaction. The raw GRIMS score is transformed into a standardized GRIMS score ranging from 1 to 9, with a cutoff point of 5. A total transformed score from 6 to 9 represents marital dissatisfaction ranging from poor to very severe (19). The Malay version of GRIMS (MVGRIMS) was used in the present study with the permission of the original author. Validation of this instrument has been done in Malaysia. The reliability of the GRIMS is 0.91 for men and 0.87 for women, with good validity under various situations (16, 20).

Statistical analysis was conducted using the Statistical Package for Social Science version 26.0 (IBM Corp., Armonk, NY, USA). Descriptive statistics of the study subjects were generated. The normality test showed that the continuous data were not normally distributed (Kolmogorov-Smirnov test, *p* < 0.05). Categorical variables were reported in frequency and percentage, while continuous variables were in median and interquartile range (IQR). Correlations between MVGRIMS score and MVFSFI total and sub-scores were measured using Spearman's correlation coefficient. MVGRIMS scores were divided into two categories. A score of ≤ 5 represented “average and above” marital satisfaction, and a score of >5 represented “poor and below” marital satisfaction. Bivariate analysis was run to examine the differences between the “average and above” group and the “poor and below” group with regards to demographic, social, and clinical characteristics, using the chi-square test or Fisher's exact test for categorical variables and Mann-Whitney *U*-test for continuous variables. Significant variables were then included in a stepwise multiple logistic regression model as independent variables to look for factors that were significantly associated with marital dissatisfaction. The logistic regression model displayed a good fit with a non-significant Hosmer-Lemeshow goodness-of-fit test (*p* = 0.730). The significance level (alpha) for all statistical tests was set at *p* < 0.05.

Ethics approval (Code: FF-2018-203) was granted by the Research Ethics Committee of the National University of Malaysia for this study before its commencement. The respondents were given options to seek professional help if they are having marital dissatisfaction and sexual dysfunction.

## Results

The sociodemographic and clinical characteristics of the study subjects are shown in Table 1. A total of 116 patients participated in this study. The median age was 59.0 years (IQR: 49.0–67.0 years); the median duration of marriage was 32 years (IQR: 20.0–40.8 years). 80.2% had a secondary and lower level of education. About two-thirds were of Malay ethnicity. The most common type of gynecologic cancer was endometrial cancer at 41.4%. Most were at the early stages of cancer according to the International Federation of Gynecology and Obstetrics (FIGO) staging system, with 50% at FIGO stage 1. The majority received either surgery alone (31.1%) or a combination of surgery and chemotherapy (30.2%) as their treatment.

**Table 1:** Characteristics of the study subjects (*N* = 116).

**Variable**	***n***	**Percentage**
Age (years)	59.0^a^	49.0–67.0^b^
Years of marriage	32.0^a^	20.0–40.8^b^
**Employment**		
Employed	29	25.0
Unemployed	27	23.3
Retired	21	18.1
Homemaker	39	33.6
**Education level**		
Primary	32	27.6
Secondary	61	52.6
Tertiary	23	19.8
**Ethnicity**		
Malay	79	68.1
Chinese	33	28.4
Indian	4	3.4
Number of children	3.0^a^	1.0–4.0^b^
**Cancer type**		
Endometrial	48	41.4
Cervical	37	31.9
Ovarian	31	26.7
**FIGO staging**		
Stage 1	58	50.0
Stage 2	21	18.1
Stage 3	30	25.9
Stage 4	7	6.0
**Treatment**		
Surgery only	36	31.1
Surgery and chemotherapy	35	30.2
Surgery and radiotherapy	19	16.4
Surgery and chemoradiotherapy	19	16.4
Chemotherapy and radiotherapy	5	4.3
Radiotherapy only	2	1.7
Years of diagnosis	3.5^a^	2.0–7.8^b^
Years of last treatment (*n* = 115)	2.0^a^	0.5–6.0^b^

a *Median*;

b *interquartile range*.

The levels of marital satisfaction among the subjects according to the MVGRIMS transformed scores are displayed in Table 2, showing a wide range of marital satisfaction. When divided into two categories, 62.1% (*n* = 72) of study subjects had average and above levels of marital satisfaction, with the remaining (37.9%, *n* = 44) reporting poor and below levels of marital satisfaction, equivalent to MVGRIMS transformed scores of >5. The transformed GRIMS scores of 1 should be treated cautiously according to the instrument's authors (16). Nonetheless, there was only one such score in the study sample. The median FSFI total score was FSFI 49.9 (IQR: 2.0–63.0). When the correlations between MVGRIMS score and MVFSFI scores were examined, the MVGRIMS score was inversely correlated with all MVFSFI sub-scores and total scores. The correlation coefficients were statistically significant, ranging from −0.286 to −0.390, indicating a moderate negative relationship between the severity of sexual dysfunction and the level of marital satisfaction (Table 3).

**Table 2:** Levels of marital satisfaction among the subjects.

**Variable**	**n**	**Percentage**
Very good	19	16.4
Good	22	19.0
Above average	15	12.9
Average	15	12.9
Poor	20	17.2
Bad	14	12.1
Severe problems	4	3.4
Very severe problems	6	5.2
Score of “1”	1	0.9

**Table 3:** Correlations between MVGRIMS transformed score and MVFSFI scores.

**Variable**	**Spearman's rho**	***p*-value**
1. MVFSFI Desire	−0.390	<0.001^*^
2. MVFSFI Arousal	−0.387	<0.001^*^
3. MVFSFI Lubrication	−0.286	0.002^*^
4. MVFSFI Orgasm	−0.342	<0.001^*^
5. MVFSFI Satisfaction	−0.367	<0.001^*^
6. MVFSFI Pain	−0.315	0.001^*^
7. MVFSFI Total Score	−0.380	<0.001^*^

* *Statistically significant*.

The subsequent bivariate analysis compared study subjects with better (average and above) and those with worse (poor and below) levels of marital satisfaction (Table 4). Older age, longer duration of the marriage, and lower MVFSFI scores were associated with worse levels of marital satisfaction. Additionally, employment status, education level, ethnicity, and cancer type were also significant. Therefore, age, duration of the marriage, MVFSFI score, employment status, education level, ethnicity, and cancer type (endometrial, cervical, or ovarian) were included in the stepwise multiple logistic regression analysis. For MVFSFI scores, only the total score was included as the sub-scores were highly correlated with each other and the total score. In the final logistic regression analysis, after controlling for confounders, lower educational levels were found to be significantly associated with poor marital satisfaction (Primary education, aOR = 12.67, 95% CI: 1.40–114.87; secondary education: aOR = 11.52, 95% CI: 1.39–95.72), while higher MVFSFI total score significantly reduced the likelihood of poor marital satisfaction (aOR = 0.979, 95% CI: 0.964–0.994) (Table 5).

**Table 4:** Comparisons between study subjects with better and with worse marital satisfaction.

**Variable**	**Marital satisfaction**				***p-*value**
	**Better**		**Worse**		
	***N***	**Percentage**	***n***	**Percentage**	
Age (years)^a^	56.5^d^	46.0–64.0^e^	63.0^d^	57.0–69.0^e^	0.001^*^
Years of marriage^a^	28.5^d^	18.0–37.0^e^	40.0^d^	28.5–44.0^e^	0.001^*^
**Employment^b^**					
Employed	25	34.7	4	9.1	0.004^*^
Unemployed	13	18.1	14	31.8	
Retired	15	20.8	6	13.6	
Homemaker	19	26.4	20	45.5	
**Education level^b^**					
Primary	14	19.4	18	40.9	<0.001^*^
Secondary	36	50.0	25	56.8	
Tertiary	22	30.6	1	2.3	
**Ethnicity^c^**					
Malay	56	77.8	23	52.3	0.017^*^
Chinese	14	19.4	19	43.2	
Indian	2	2.8	2	4.5	
Number of childrena	2.5^d^	1.0–4.0^e^	3.0^d^	1.3–4.0^e^	0.249
**Cancer type^b^**					
Endometrial	28	38.9	20	45.5	0.036^*^
Cervical	19	26.4	18	40.9	
Ovarian	25	34.7	6	13.6	
**FIGO staging^c^**					
Stage 1	35	48.6	23	52.3	0.757
Stage 2	12	16.7	9	20.5	
Stage 3	21	29.2	9	20.5	
Stage 4	4	5.6	3	6.8	
**Treatment^c^**					
Surgery only	22	30.6	14	31.8	0.112
Surgery and chemotherapy	27	37.5	8	18.2	
Surgery and radiotherapy	10	13.9	9	20.5	
Surgery and chemoradiotherapy	10	13.9	9	20.5	
Chemotherapy and radiotherapy	3	4.2	2	4.5	
Radiotherapy only	0	0.0	2	4.5	
Years of diagnosisa	4.0^d^	2.0–7.0^e^	2.5^d^	2.0–8.0^e^	0.62
Years of last treatmenta	2.0^d^	0.4–6.0^e^	2.0^d^	0.6–5.8^e^	0.947
**MVFSFI scores**					
Desire	5.0^d^	3.0–6.0^e^	2.0^d^	2.0–4.0^e^	<0.001^*^
Arousal	12.0^d^	1.0–14.0^e^	0.0^d^	0.0–7.8^e^	<0.001^*^
Lubrication	11.0^d^	0.0–15.0^e^	0.0^d^	0.0–10.0^e^	<0.001^*^
Orgasm	9.0^d^	0.0–11.0^e^	0.0^d^	0.0–7.0^e^	<0.001^*^
Satisfaction	10.0^d^	0.0–12.8^e^	0.0^d^	0.0–7.8^e^	<0.001^*^
Pain	9.0^d^	0.0–12.0^e^	0.0^d^	0.0–9.0^e^	0.001^*^
Total	57.5^d^	5.3–68.3^e^	2.0^d^	2.0–50.5^e^	<0.001^*^

a *Mann-Whitney U test*;

b *Chi-squared test*;

c *Fisher's exact test*;

d *median*;

e *interquartile range*.

* *Statistically significant*.

**Table 5:** Logistic regression analysis for factors associated with worse marital satisfaction.

**Variable**	**Adjusted OR**	**95% CI**		***p* value**
		**Lower**	**Upper**	
Years of marriage	1.03	0.99	1.07	0.102
**Education level**				
Primary	12.67	1.40	114.87	0.024^*^
Secondary	11.52	1.39	95.72	0.024^*^
Tertiary	1.00			
FSFI Total Score	0.979	0.964	0.994	0.007^*^

*χ^2^ = 32.662, df = 5, p <0.001; Nagelkerke R^2^ = 0.337*.

* *Statistically significant*.

*Post-hoc* analyses were conducted to test whether the inverse association between sexual dysfunction and marital satisfaction only occurred among study subjects with lower educational levels (secondary and below) and not those with tertiary education. A statistically significant correlation was found between MVFSFI total score and MVGRIMS score among participants with primary and secondary education, Spearman's rho = −0.350 (*p* = 0.001). Nonetheless, the Spearman's correlation coefficient between FSFI total score and GRIMS score among participants with tertiary education was not significant (Spearman's rho = −0.166, *p* = 0.450). To further investigate the possibility of education level moderating the relationship between sexual dysfunction and marital dissatisfaction, a moderated regression analysis was performed using PROCESS version 3.5. The outcome variable for analysis was MVGRIMS transformed score. The predictor variable was FSFI total score. The moderator variable was education level. The interaction between FSFI total score and education level was statistically insignificant (B = 0.0088, 95% CI: −0.0075 to 0.0252, *p* = 0.2856). Hence, education level did not moderate the relationship between sexual dysfunction and marital dissatisfaction. Education level and sexual dysfunction were independently associated with marital dissatisfaction.

## Discussion

In this study, we found that lower levels of education and lower MVFSFI scores (indicating greater degrees of sexual dysfunction) were significant factors associated with poor marital satisfaction, as defined by an MVGRIMS transformed score of >5. Furthermore, additional analyses confirmed that education level and sexual dysfunction had distinct associations with marital dissatisfaction.

Our finding that sexual dysfunction was associated significantly with martial dissatisfaction agrees with the previous study by (5) among breast and genital cancer patients. They found that patients who fulfilled the criteria of sexual dysfunction had significantly lower mean scores for quality of relationship with spouse compared with those without sexual dysfunction. It is speculated that the relationship between sexual function and marital satisfaction among cancer patients can be two-way. Women with a closer relationship with their spouses may have a better sex life with their husbands, thereby reporting less sexual dysfunction (6). Conversely, due to the impact of the illness and the consequences of treatment, patients may experience emotional and physical symptoms that interfere with their sexual desire and sexual function, resulting in reduced sexual intercourse with their husbands. These symptoms may consequently contribute to relationship problems and marital dissatisfaction (21). A study by Fahami et al. (5) found that the quality of a marital relationship is one of the crucial factors predicting sexual functioning, which varies among different types of cancers. A correlational study among more than 100 patients with breast and gynecological cancers concluded that there was a significant correlation between sexual functioning and quality of marital relationship. For couples who are longer in a relationship, as seen in the long median duration of marriage of 32 years in our study, addressing both issues on marital and sexual relationship is important for a better quality of life and health care.

The prevalence of female sexual dysfunction among patients with a gynecologic cancer was as high as 78.44 % (95 % CI 68.36–88.52%) as measured using the FSFI in a meta-analysis (22). Treatments of gynecologic cancers are prone to cause sexual dysfunction (23). Surgical procedures for gynecologic cancers may result in body image changes, pain, and problem in attaining orgasm (24), or even premature menopausal symptoms (25). Chemotherapy may further impair patients' appearance with alopecia and weight loss (26), further contributing to sexual dysfunction. Likewise, radiotherapy may lead to vaginal stenosis and dyspareunia (27). Thus, sexual dysfunction can result in a substantial deleterious impact on marital relationships.

Education level has been demonstrated to be associated with female sexual dysfunction. In a large Iranian study involving 1,409 women in the general population, lower educational level was identified as a significant factor across different domains of sexual dysfunction including desire, arousal, orgasm, lubrication, and satisfaction with odds ratios ranging from 1.80 to 4.01 (28). Given the high likelihood of sexual dysfunction among gynecological cancer survivors (22, 23), it is probable that low education level also contributes considerably to poor adjustment of sexual function post-treatment, just as how it affects psychosocial adjustment in general among cancer survivors (9). For future interventions, the inclusion of patient's educational programs and relationship/couple therapy during rehabilitation programs is pivotal to enhance the couple's quality of marital relationship and subsequent sexual functioning among patients with gynecological cancer. Interestingly, studies have shown that a relationship between marital and sexual satisfaction may change over time. It is believed that the temporal association between these outcomes is dynamic rather than static (29). Moreover, patient education involving communications between patients and clinicians regarding sexual experience following treatment, alongside psychological interventions such as cognitive therapy, is important in improving the sexual well-being of the patients (30).

Studies have demonstrated previously that various demographic factors affect marital satisfaction. Such factors include age, number of children, length of the marriage, and educational attainment (31, 32). In this study, we did not find that age, the number of children, and length of marriage associate with marital satisfaction after controlling for confounders. It is interesting to note that in the study by Jose and Alfons among adults in the general population, education level was correlated with general-life adjustment problems and not sexual adjustment in the marital satisfaction questionnaire they used (Maudsley Marital Questionnaire, MMQ) (31). Specifically for women, there is also conflicting but limited evidence regarding the relationship between education level and marital satisfaction. An earlier study found that women with more advanced education were more likely to experience unstable marriages (33). Contrarily, a population study in the United States concluded that more highly educated women had lower rates of marital dissolution (34). More research to study the influence of education level on marital satisfaction, especially among gynecologic cancer survivors, is thus needed.

Given that sexuality is an integral part of the quality of life, it is essential that health experts actively disclose information to women and their partners about the consequences of treatment in gynecologic cancers (35–38). It is crucial to authorize and normalize sexuality in this context of continuous consultation, for example, addressing their queries about their health and sexual-related matters during the routine consultation in the clinic. Future study in gynecologic cancer and sexuality demands researchers to address the pivotal role of bio-psycho-social interactions—how body and mind interact. For example, malaise due to the complication of radiotherapy causes low sexual desire, and low sexual desire may cause a lack of sexual satisfaction. This understanding and practice are often neglected in gynecological consultations with patients and healthcare workers (39). These include the physical/anatomical/material effects of disease treatments, sexual intimacy, psychodynamic factors, and their relationship with the partner. Shaping women's experiences and practices of sexuality post-gynecologic cancer can be a significant challenge to many clinicians.

This study had some limitations. Recruitment of study subjects by convenience sampling at a single site might limit the generalizability of its findings. However, as the study site is one of the few tertiary centers for gynecologic cancer treatment in the country, it is still quite representative of the Malaysian study population. As the study was cross-sectional in nature, the temporal relationship between studied variables, such as sexual dysfunction and marital dissatisfaction, could not be elucidated. Recall bias when answering the questionnaires was also possible.

In conclusion, both sexual dysfunction and education level are associated with marital satisfaction among gynecologic cancer survivors. Sexual dysfunction and low education level among gynecologic cancer survivors may independently affect their marital satisfaction. Given this, targeted efforts focusing on patients with low education levels and sexual dysfunction may help to improve their marital life.

## Data Availability Statement

The original contributions presented in the study are included in the article/supplementary material, further inquiries can be directed to the corresponding author.

## Ethics Statement

The studies involving human participants were reviewed and approved by Research Ethics Committee, National University of Malaysia. The patients/participants provided their written informed consent to participate in this study.

## Author Contributions

SM and SH conceived and designed the study, reviewed and interpreted the results, and assisted in the writing of the manuscript. NN, SM, SH, and LW were involved in data collection. LW analyzed the data and wrote the main manuscript text. All authors contributed to the article and approved the submitted version.

## Conflict of Interest

The authors declare that the research was conducted in the absence of any commercial or financial relationships that could be construed as a potential conflict of interest.

## Publisher's Note

All claims expressed in this article are solely those of the authors and do not necessarily represent those of their affiliated organizations, or those of the publisher, the editors and the reviewers. Any product that may be evaluated in this article, or claim that may be made by its manufacturer, is not guaranteed or endorsed by the publisher.
